# Management of cow’s milk protein allergy in primary care

**DOI:** 10.1186/2045-7022-1-S1-P40

**Published:** 2011-08-12

**Authors:** Dinkar Bakshi, Ian Pollock

**Affiliations:** 1 Homerton University Hospital, London, UK; 2 Chase Farm Hospital, Paediatrics, London, UK

## Background

The incidence of Cow's Milk Protein Allergy ( CMPA ) is increasing in the United Kingdom. However, many practitioners in primary care are unsure about its diagnosis and management. There is usually a lag period of a few months, between the first presentation of children with CMPA to their General Practitioner ( GP ), and referral to an Allergist. Enfield area comprises a middle-class, and relatively affluent population of London.

## Aim

To identify the factors that influence CMPA primary care management and referral practice, in Enfield area in London.

## Methods

A web-based questionnaire was completed by 46 General Practitioners in Enfield area, in January, 2010.

## Results

87% of the respondents felt that children with suspected CMPA, should be seen by an Allergist within 4 weeks from the date of referral. 54.3% of the GPs said that they referred children to a Paediatric Allergist, after a trial of extensively hydrolysed or aminoacid formula milk. The most popular milk substitutes in primary care in Enfield are Nutramigen (60.9%), Soya milk (52.2%), Neocate (47.8%) and Peptijunior (17.4%). 82.6% GPs said they prescribe Epipen (Adrenaline autoinjector) for life threatening allergic symptoms. With regards to referral to a Paediatric Dietitian, 34.8% GPs said they refer less than 25% cases of CMPA, whereas 43.5% GPs refer over 75% children with CMPA. GPs got information about management of CMPA primarily from Paediatricians (71.7%), while a significant number also accessed journals (52.2%) and the internet (50.0%).

## Conclusion

The management of CMPA in primary care in Enfield is not consistent, and dependent upon the individual GPs. We have initiated the formulation and dissemination of a local guideline, to enable practitioners in primary care to refer children with suspected CMPA to the Allergy clinic.

